# Commentary: Beyond the face: how context modulates emotion processing in frontotemporal dementia subtypes

**DOI:** 10.3389/fnagi.2020.00244

**Published:** 2020-08-20

**Authors:** Liang Chen, Xu Chen

**Affiliations:** ^1^Faculty of Psychology, Southwest University, Chongqing, China; ^2^Research Center of Mental Health Education, Southwest University, Chongqing, China

**Keywords:** facial expressions, bodily expressions, emotion recognition, frontotemporal dementia, neuroimaging

The first case of language variant of frontotemporal dementia (FTD) was reported by Pick (1892), in which an autopsy revealed focal atrophy of the temporal lobe. Mesulam (1982) reported six patients with a slowly progressing aphasic disorder later called “primary progressive aphasia.” The lesion was located at the site of the focal left perisylvian or temporal lobe, but these patients did not have global symptoms of dementia, including additional intellectual and behavioral disturbances. Some separate cases of right frontal or temporal degeneration were subsequently reported, as patients did not recognize their family (i.e., prosopagnosia), nor did they remember spatial topography. A consensus of “frontotemporal lobar degeneration” was put forth by Neary et al. (1998) and Snowden et al. (2002). Recently, the updated relevant diagnostic criteria and classification system with greater sensitivity have also been proposed (Gorno-Tempini et al., 2011; Rascovsky et al., 2011). Evidence from epidemiological studies has shown that FTD is the most common type of primary degenerative dementia, after Alzheimer's disease and dementia with Lewy bodies, accounting for about 20% of presenile dementia (Ratnavalli et al., 2002; Snowden et al., 2002; Coyle-Gilchrist et al., 2016). Two clinical subtypes of FTD are recognized in the literature. The first clinical subtype of FTD is called behavioral variant frontotemporal dementia (bvFTD), in which the most pronounced brain atrophy is in the mesial and orbitofrontal regions and also includes the temporal lobe (Rosen et al., 2002a, 2005; Peters et al., 2006; Whitwell et al., 2009). In some patients, the alteration of limbic network has been observed (e.g., the insula, striatum, anterior cingulate, and amygdala) (Boccardi et al., 2005; Rascovsky et al., 2011). Their primary motor cortex generally remains intact (Whitwell et al., 2009). The patients with bvFTD have some key clinical characteristics (e.g., disinhibition, apathy, loss of empathy, compulsive behaviors, executive dysfunction) (Rascovsky et al., 2011). The second clinical subtype of FTD is semantic dementia (SD) that presents the defining feature of impairments in naming objects, word comprehension, and other conceptual or semantic knowledge (Hodges et al., 1992; Landin-Romero et al., 2016). The left-lateralized anterior temporal lobe is the primary region of atrophy in SD. In addition, degeneration of the temporal and limbic areas is also reported (Mummery et al., 2000; Yang et al., 2012). Note that about 30% of cases, however, present with predominantly right-lateralized brain atrophy, which is accompanied by various behavioral symptoms (e.g., social disinhibition, emotion recognition, and aggression) and emotional changes (e.g., depression) (Chan et al., 2009; Kumfor et al., 2016).

Social cognitive impairment exists in both bvFTD and SD. In daily life, social cognition typically involves being able to identify others' states correctly, especially their emotional state based on facial expressions. Given these cognitive and neurological abnormalities mentioned above, people with bvFTD and SD have impaired processing of emotions (Kumfor and Piguet, 2012). Patients with both bvFTD and SD show disrupted recognition of basic negative facial expressions (i.e., sad, fearful, disgust, and angry) compared with healthy controls. However, they can recognize happy facial expressions, which involves dissociable brain regions (Kumfor et al., 2013). The recognition of happy faces generally intact in bvFTD, but this pattern in SD relies on the test stimuli used (Kumfor and Piguet, 2012). The identification accuracy of emotional faces in terms of social cognition and emotional assessment significantly relates to gray matter volume in the ventromedial prefrontal cortex in bvFTD (Bertoux et al., 2012). FTD also shows lower performance in tracking dynamic emotional stimuli, which is primarily associated with gray matter loss in the right lateral orbitofrontal cortex (Goodkind et al., 2012). These neurological abnormalities further impair the patient's future thinking, empathy, and theory of mind (Landin-Romero et al., 2016).

However, previous studies have mainly focused on isolated emotional cues (e.g., face, voice), and these stimuli do not encompass the complete range of human emotions (Aviezer et al., 2012b, 2017). Recent studies have demonstrated the importance of context in discriminating between facial expressions. Meeren et al. (2005) first discovered the automatic integration of facial expressions and bodily expressions in an early time window (~115 ms), using event-related potentials technique. Moreover, the categorization of facial expressions (e.g., angry, disgust, sad, and fearful) depends on their accompanied bodily expressions (Aviezer et al., 2008). This effect occurs not only for facial expressions that convey approximate values (e.g., angry and disgust) but also for expressions that convey significant differences in values (e.g., win and lose) (Aviezer et al., 2012a). Although deficits in facial emotion recognition in both bvFTD and SD have been thoroughly investigated (Kumfor and Piguet, 2012), it is unclear whether contextual stimuli facilitate or enhance emotion recognition in these patients. Previous research has indirectly investigated the difficulty in understanding contextual information in patients with FTD. For example, bvFTD patients show abnormalities in interpreting sarcastic, but not sincere statements, which is linked to the disproportionate atrophy in the right amygdala, right lateral orbitofrontal cortex, right temporal pole (Kipps et al., 2009), and precuneus (Kumfor et al., 2017). Findings of Kipps et al. (2009) and Kumfor et al. (2017) may be explained as dysfunctions in integrating discordant contextual cues (e.g., voice and face) to judge the true intent of others (Ibanez and Manes, 2012). The social context network model (SCNM) has recently been proposed (Ibanez and Manes, 2012; Baez et al., 2017), which holds that social situations are commonly context-embedded. The integration of target and contextual information is underpinned by a frontal-insular-temporal network: (a) updating context cues to make predictions by the involvement of several frontal areas (i.e., orbitofrontal cortex, lateral prefrontal cortex, and superior orbital sulcus), (b) interaction between internal and external milieus in the insular cortex, and (c) context-target associative learning in the temporal lobe. The SCNM describes the deficits of social cognition (e.g., decision-making, figurative language, facial recognition, empathy, and theory of mind) in bvFTD patients as context impairments. Note that emotional integration and the core atrophic areas in FTD patients feature overlap in their underlying neuroanatomical correlates (e.g., the frontal and temporal areas, limbic network) (de Gelder, 2006; Van den Stock et al., 2014; Hortensius et al., 2016).

These topics were addressed in a recent study published in the journal of *Brain*; Kumfor and colleagues conducted a series of neuropsychological tests, emotion processing tasks, and a whole-brain structural MRI. First, a pre-study neuropsychological assessment was conducted. Second, all participants completed tasks A-C: recognition of isolated facial expressions (i.e., face only) in task A; recognition of bodily context (i.e., body language only) in task B; contextual effects of body language on the face in task C. Similar to previous studies (e.g., Aviezer et al., 2008), these tasks focused only on the emotions of anger, disgust, fear, and sadness. Categorization accuracy in tasks A and B were calculated, along with contextual influence (i.e., the percentage of times faces were identified as expressing the bodily context emotion) from task C. Finally, high-resolution T1 structural images were acquired. A total of 51 subjects (19 with bvFTD, 12 with SD, and 20 controls) participated in all the tests.

Their results revealed the following: first, over-reliance on contextual information appeared in people with bvFTD. Specifically, in recognition of isolated emotions from facial and body language cues (i.e., tasks A and B), people with bvFTD and SD showed similar lower accuracy than did the controls. While categorizing facial expressions in incongruent contexts, those with bvFTD showed worse accuracy and were more likely to be deceived by bodily stimuli than were both people with SD and the controls. Second, there was a similar response pattern in contextual influence between people with SD and the control group. The perceived similarity between facial expressions and contextual emotion could be a crucial determinant of incongruent face-body pairings in the SD group, as well as in the controls. For instance, an angry face was roughly similar to a disgusted face. Thus, when a disgusted body was presented as context, the face was labeled as angry. Third, the right parahippocampal/amygdala and left precentral gyrus were associated with categorization accuracy and contextual influence in people with bvFTD and those with SD. Generally, the lower integrity between these two regions related to a higher degree of contextual influence. Moreover, lower integrity of the bilateral temporal fusiform cortex and orbitofrontal cortex was also observed in bvFTD. Although the left precentral gyrus is not typically reported to be involved in facial expression processing, its increased activation has been found when viewing bodily images (Kourtzi and Kanwisher, 2000), gestures, actions, and implied motion (Saggar et al., 2014; Kolesar et al., 2017).

Kumfor et al. (2018)'s findings offer a unique opportunity to demonstrate the relative contributions of the right parahippocampal/amygdala and left precentral gyrus for the interaction of contextual information and emotion processing. While a recent study with a group that underwent unilateral anterior temporal lobe resections, including the amygdala, has suggested that the amygdala and anterior temporal lobe are not necessary for recognition of dynamic bodily expressions (Van de Vliet et al., 2018), Kumfor et al. (2018) explored a more real-life situation, that is, emotion recognition with contextual information. The greater contextual influence in bvFTD patients reflects difficulty in integrating and modulating emotional contextual information when recognizing the target, which might be akin to the ‘environmental dependency syndrome’ described in frontal lesion patients (Lhermitte, 1983, 1986; Lhermitte et al., 1986). Specifically, individuals could have inappropriate behaviors that are induced by external clues or cues, regardless of the intended goals and emotional consequences. The findings of Kumfor et al. (2018) also supports the SCNM. The frontal lobe lesions might result in the activation of automatic reliance on the emotional context when recognizing the target (e.g., facial expression) by the external clues (e.g., bodily expression) in bvFTD patients. In the SCNM, frontal areas are in charge of updating context cues to make predictions. Kumfor et al. (2018)'s results suggest that contextual influence is sensitive to social cognition impairment and is specific to bvFTD patients. However, unlike bvFTD patients, SD patients' performance in the task C is modulated by context, indicating a degree of contextual integration. The SCNM proposes that temporal lobe is responsible for context-target associative learning, via basic related process with the perirhinal cortex, hippocampus, and amygdala. Kumfor et al. (2018) also revealed that abnormal contextual influence was associated with lower integrity of the right parahippocampal gyrus/amygdala and left precentral gyrus. Therefore, these behavioral and neuroimaging findings are in accordance with the SCNM.

Moreover, we suggest some clinical implications here. Early emotional blunting, which refers to an inappropriate emotional shallowness with unconcern and a loss of emotional warmth, empathy, sympathy, and an indifference to others, is one of the core diagnostic features in people with FTD (Gorno-Tempini et al., 2011; Rascovsky et al., 2011). The difficulty in facial expressions recognition has also been thoroughly investigated in both people with bvFTD and SD (Lavenu et al., 1999; Rosen et al., 2002b; Kumfor and Piguet, 2012; Kumfor et al., 2013). The different response patterns (i.e., over-reliance on contextual information observed in bvFTD, rather than SD) might provide a relative reference for diagnosing subtypes of global FTD and enrich our understanding of social cognition impairment in these groups. Future research could also build on the contextual emotion recognition task, use more technical measures, and develop multimodal biomarkers for diagnosis of FTD including, but not limited to, whole-brain structural MRI and behavioral performance. Facial expressiveness and physiological responses (e.g., skin conductance level, SCL) in FTD when processing emotional stimuli are worth considering. For instance, in Kumfor et al. (2019), bvFTD patients, SD patients, and healthy controls were required to passively view emotional video clips, and surface facial electromyography (EMG) and SCL were recorded. SD patients showed increased zygomaticus reactions to neutral and positive videos, but bvFTD had the same facial EMG responses in all conditions. Both FTD patient groups did not show greater SCL when viewing emotional videos, unlike controls. Patients with bvFTD also have difficulties to imitate facial expressions (Gola et al., 2017). Note that simultaneous heart rate and pupillometric responses are meaningful as well, because different subtypes of FTD may have slight differences in these two indicators when processing complex socio-emotional stimuli. For example, Marshall et al. (2019) performed a task fMRI with simultaneous heart rate and pupillometric recordings, during which four groups (bvFTD; semantic variant primary progressive aphasia, svPPA; non-fluent variant primary progressive aphasia, nvPPA; healthy controls) were required to passively view dynamic universal emotional facial expressions. They found that, compared to healthy controls, all syndromes showed significant impairments in cardiac reactivity, and only individuals with nvPPA had impaired pupillary reactivity. They also revealed some syndrome-specific activations to predict facial emotion recognition accuracy (e.g., anterior insula and caudate for bvFTD, anterior temporal cortex for svPPA). Therefore, the recent studies provide grounds for future researchers to incorporate technical measures to systematically develop the multimodal biomarkers of FTD in contextual emotion recognition. In addition, future interventions might reduce over-reliance on contextual information in people with bvFTD through priming or subliminal presentation. Furthermore, congruent contextual information could be provided during treatment to improve the patients' abilities regarding emotion recognition in both those with bvFTD and SD. These hypotheses will be examined in the future.

In summary, the authors (Kumfor et al., 2018) provide theoretically and clinically significant evidence of how contextual information modulates emotion recognition in the FTD subtypes. Future research is warranted to expand the sample size of patients with SD to improve statistical power, especially for right-lateralized SD patients. Moreover, it is needed to recruit a large sample of both healthy adults and neurological patients to validate the three tasks into a clinically sensitive test of contextual emotion recognition, like the classical TASIT (Henry et al., 2016). Note that some details of these three tasks should be improved. For example, the task A should display one face, rather than an array of seven faces, and use labeling paradigm, which may be comparable with tasks B and C for task demand; more basic or complex emotions (e.g., happy, pride) could be included to expand our understanding of social cognition in neurological disorders. Finally, other contextual cues from multiple modalities should be explored, such as scene and voice. Patients with bvFTD are also poor at classifying laughter, retching, crying (Keane et al., 2002), and emotional music (Hsieh et al., 2011, 2012; Fittipaldi et al., 2019). Emotional integration and conflict in healthy people have been widely investigated (Schirmer and Adolphs, 2017), but that in the FTD group is unclear. Findings of Kumfor et al. (2018) are likely to inspire more insights and therapeutic interventions regarding social cognition impairment.

## Author Contributions

All authors listed have made a substantial, direct and intellectual contribution to the work, and approved it for publication.

## Conflict of Interest

The authors declare that the research was conducted in the absence of any commercial or financial relationships that could be construed as a potential conflict of interest.
